# Novel perspectives on the therapeutic role of cryptotanshinone in the management of stem cell behaviors for high-incidence diseases

**DOI:** 10.3389/fphar.2022.971444

**Published:** 2022-08-15

**Authors:** Xiaomeng Guo, Ruishuang Ma, Meng Wang, Benson Wui-Man Lau, Xiaopeng Chen, Yue Li

**Affiliations:** ^1^ State Key Laboratory of Component-Based Chinese Medicine, Institute of Traditional Chinese Medicine, Tianjin University of Traditional Chinese Medicine, Tianjin, China; ^2^ Department of Rehabilitation Sciences, The Hong Kong Polytechnic University, Hong Kong, Hong Kong SAR, China

**Keywords:** cryptotanshinone, high-incidence diseases, proliferation, differentiation, apoptosis, stem cell, self-renewal

## Abstract

Cryptotanshinone (CTS), a diterpenoid quinone, is found mostly in *Salvia miltiorrhiza* Bunge (*S. miltiorrhiza*) and plays a crucial role in many cellular processes, such as cell proliferation/self-renewal, differentiation and apoptosis. In particular, CTS’s profound physiological impact on various stem cell populations and their maintenance and fate determination could improve the efficiency and accuracy of stem cell therapy for high-incidence disease. However, as much promise CTS holds, these CTS-mediated processes are complex and multifactorial and many of the underlying mechanisms as well as their clinical significance for high-incidence diseases are not yet fully understood. This review aims to shed light on the impact and mechanisms of CTS on the actions of diverse stem cells and the involvement of CTS in the many processes of stem cell behavior and provide new insights for the application of CTS and stem cell therapy in treating high-incidence diseases.

## Graphical Abstract

This review summarizes the regulatory role of CTS in the various behaviors of different stem cell populations, and highlights the potential of CST and stem cell therapy in high-incidence diseases.

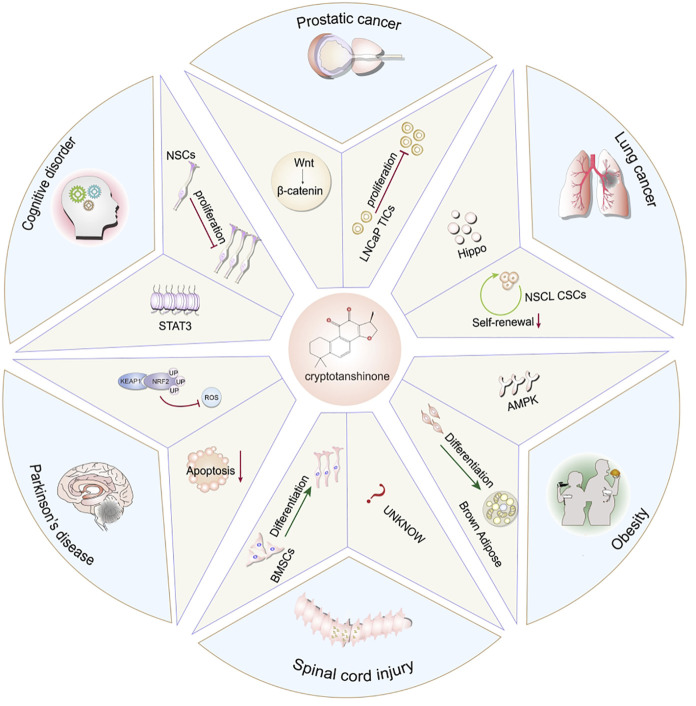

## Introduction

*S. miltiorrhiza* is a well-known traditional Chinese herb utilized as a medicine and a health-promoting food (Shi et al., 2019). Cryptotanshinone is a natural product found in *S. miltiorrhiza*. Many compound medicines containing CTS are currently available on the Chinese market, including Tanshinone Capsules, Danhong Injection (Wang et al., 2021c), and Compound Danshen Dropping Pills (Wang et al., 2022b). In addition, the clinical trials of chemical drugs are summarized (Table 1). CTS is being studied for a variety of pharmacological effects, including anti-inflammatory (Wu et al., 2020; Liu et al., 2021a), neuroprotective (Mao et al., 2021), cardioprotective (Wang et al., 2021e), anti-fibrosis (Lo et al., 2017; Zhang et al., 2020) and anti-tumor (Dong et al., 2018; Han et al., 2019; Vundavilli et al., 2022). The diversity of pharmacological effects of CTS demonstrates unique potential for the treatment of high-incidence diseases.

**TABLE 1:** Clinical trials of compound medicines containing CTS in various diseases (www.clinicaltrials.gov).

Conditions	Age	Population	Drug Names	Duration (weeks)	Status	ClinicalTrials.gov Identifier	Website Links	References
Covid-19	Child, Adult, Older Adult	Intermediate-size	T89 capsule	2	Available	NCT04646031	https://clinicaltrials.gov/ct2/show/NCT04646031?term=danshen&draw=4&rank=22	N/A
Acute Mountain Sickness	18–50	58	CDDP	1	Completed	NCT03270787	https://clinicaltrials.gov/ct2/show/NCT03270787?term=danshen&recrs=e&draw=2&rank=1	N/A
Myocardial Infarction	18–75	268	CDDP	N/A	Recruiting	NCT05000411	https://clinicaltrials.gov/ct2/show/NCT05000411?term=danshen&draw=2&rank=9	N/A
Hypertension	20–55	20	T89 capsule	4	Completed	NCT01679028	https://clinicaltrials.gov/ct2/show/NCT01679028?term=danshen&draw=3&rank=26	N/A
Angina Pectoris	20–80	1,004	T89 capsule	4	Completed	NCT01659580	https://clinicaltrials.gov/ct2/show/NCT01659580?term=Salvia+miltiorrhiza&draw=2&rank=25	Shi et al. (2020)
Stable Angina	18–50	24	T89 capsule	4	Completed	NCT01473888	https://clinicaltrials.gov/ct2/show/NCT01473888?term=T89&draw=2&rank=4	N/A
Acute Mountain Sickness (AMS)	18–55	132	T89 capsule	2	Completed	NCT03552263	https://clinicaltrials.gov/ct2/show/NCT03552263?term=danshen&recrs=e&draw=2&rank=16	N/A
Angina Pectoris	18–80	124	T89 capsule	12	Completed	NCT00797953	https://pubmed.ncbi.nlm.nih.gov/?term=NCT00797953&filter=simsearch1.fha	N/A
Unstable Angina Pectoris	35–75	160	Danhong injection	4	Completed	NCT02007187	https://clinicaltrials.gov/ct2/show/NCT02007187?term=danshen&draw=3&rank=46	Chen et al. (2022)
Acute Stroke	18–70	1,503	Danhong injection	13	Completed	NCT01677208	https://clinicaltrials.gov/ct2/show/NCT01677208?term=danshen&recrs=e&draw=2&rank=23	Li et al. (2015)
Chronic Stable Angina	18–70	920	Danhong injection	13	Completed	NCT01681316	https://clinicaltrials.gov/ct2/show/NCT01681316?term=danshen&draw=3&rank=47	Wang et al. (2015a), Liu et al. (2021b)
Fatty Liver Disease	18–65	118	Tablet *salviae miltiorrhiza*e	24	Active, not recruiting	NCT05076058	https://clinicaltrials.gov/ct2/show/NCT05076058?term=danshen&draw=2&rank=11	N/A
Peripheral Arterial Disease Intermittent Claudication	40 Years and older	107	Danshen Gegen Capsule	24	Completed	NCT02380794	https://clinicaltrials.gov/ct2/show/NCT02380794?term=danshen&draw=8&rank=5	N/A

In recent years, research on some high-incidence diseases, such as neurodegenerative diseases, cancer, obesity and obesity-related complications has increased (Blüher, 2019; Siegel et al., 2022). One is that these diseases exist in all populations and age stages, the other is that these diseases have a high mortality rate (Hao et al., 2021; Gribsholt et al., 2022; Siegel et al., 2022), and because of the complexity and intractability of these diseases, the effect of treatment is limited. In contrast, the ability of CTS to modulate different signaling pathways may offer therapeutic and preventive benefits. CTS has been shown to be effective in treating these high-incidence diseases such as obesity, diabetes (Kim et al., 2007), atherosclerosis (Hao et al., 2019), neurodegenerative disease (Maione et al., 2018) and cancer (Chen et al., 2021; Shi et al., 2022). But its effects on stem cell behaviors remain unclear. CTS, as diterpenoid quinones, mostly have o-quinone or para-quinone structures with ternary or four-membered carbon rings on the skeleton (Wang et al., 2017). As lipid soluble components, CTS can more easily pass through the cell membrane (Chen et al., 2012). In addition, diterpenoid quinones can determine the fate of stem cells through a variety of different mechanisms (Li et al., 2018a; Kim et al., 2019; Liu et al., 2019). It provides a further opportunity and reference for future CTS research. Our work focuses on the diverse and critical roles of CTS in various types of stem cells, and the potential of stem cell-dependent therapy for high-incidence diseases.

Stem cells can be extracted from embryonic and postnatal animal tissues and have the dual properties of self-renewal and differentiation (De Los Angeles et al., 2015). Stem cell research has made significant progress in recent years due to its unique features that hold great promise for medicine (Madl et al., 2018). Stem cell transplantation, in which damaged tissues or organs can be healed via autologous or allogeneic stem cell transplantation, is one of the growing subjects of interest in this discipline. Based on the qualities of stem cells listed above, stem cell therapies have been widely employed in osteoporosis and obesity and intensively explored in neurodegenerative diseases and cancer (Lunn et al., 2011; Plaks et al., 2015; Pagnotti et al., 2019). Nevertheless, stem cell therapy has disadvantages, including a low transplantation rate and a low survival rate (Vissers et al., 2019; Xu et al., 2019). Combining stem cell therapy with Chinese herbs may improve stem cell therapy efficacy. CTS, for example, can enhance the development of C3H10T1/2 mesenchymal stem cells (C3H10T1/2 MSCs) into brown adipocytes (Imran et al., 2017). CTS can also enhance the development of bone marrow mesenchymal stem cells (BMSCs) into neural lineage cells (Deng et al., 2006), which may open up new avenues of investigation for obesity and spinal injuries.

To demonstrate the multifunctional potential of CTS as a treatment for high-incidence diseases, this article reviews its regulatory effects on C3H101/2 MSCs, BMSCs, cancer stem cells (CSCs), and neural stem/progenitor cells (NSCs/NPCs) (Table 2), and discusses the mechanism by which CTS promotes cell proliferation/self-renewal and differentiation. This review demonstrates the potential for CTS to improve the application of stem cell therapies and stem cell transplantation and provide new therapeutic strategies for the high-incidence diseases.

**TABLE 2:** The alternations and influences of CTS on physiological behavior of various stem cells.

Cell Types	Species	Phenotypes	DOSE	Molecular target/mechanisms	References
C3H10T1/2 MSC	Mouse	Cell differentiation	8 µM	P38-MAPK/AMPKα/Smad1/5	Imran et al. (2017)
BMSC	Monkey	Cell differentiation	10 μg/ml	Unknown pathways	Deng et al. (2006)
NSC	Human	Cell proliferation	Unknow	STAT3	Zhang et al. (2016a)
NPC	Human	Cell apoptosis	1 and 3 µM	NRF2	Lee et al. (2020)
NSCLC CSC	Human	Cell self-renewal	5–20 µM	Hippo	Jin et al. (2020)
LNCaP TIC	Human	Cell self-renewal and Proliferation	2.5, 5 and 10 µM	Wnt/β-catenin	Zhang et al. (2016b)

## CTS and C3H10T1/2 mesenchymal stem cells

**FIGURE 1:**
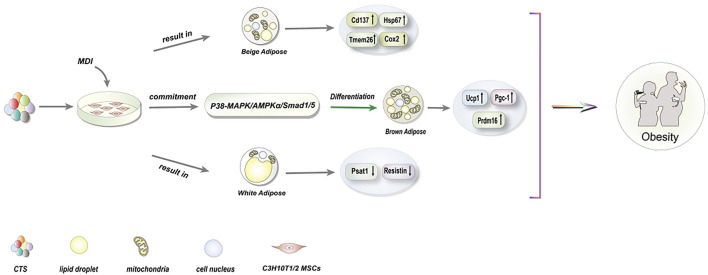
Schematic diagrams of the effects of CTS on differentiation of C3H10T1/2 MSCs for the treatment of obesity.

C3H10T1/2 can be used as a viable MSC model to determine the ability of potentially obese cells to differentiate into adipocytes (Wen et al., 2022). The induction of C3H10T1/2 MSCs into brown adipocytes is critical for obesity treatment. Between the mid-1970s and 2020, the World Health Organization estimates that the worldwide obesity rate has tripled and may continue to rise (Kumanyika and Dietz, 2020). Obesity is strongly associated with cardiovascular disease, type 2 diabetes, and cancer, the latter of which accounts for most human health problems (GBD 2015 Obesity Collaborators et al., 2017; Kumanyika and Dietz, 2020). Intriguingly, CTS has been revealed to induce differentiation of C3H10T1/2 MSCs into adipocyte lineages (Figure 1). Understanding the mechanism by which CTS induces the differentiation of C3H10T1/2 MSCs is critical for treating obesity.

CTS acts as an anti-diabetic and anti-obesity agent by stimulating the amp-activated protein kinase (AMPK) (Kim et al., 2007). AMPK is required for metabolic management and obesity-related disorders (Lopez, 2017; Garcia et al., 2019) since it regulates brown adipose tissue (BAT) thermogenesis and white adipose tissue browning (Lopez and Tena-Sempere, 2017). Recently, one appealing technique for combating obesity has been the use of heat generated by BAT and beige adipose tissue to treat obesity (Lu et al., 2016). CTS stimulates brown cell development in C3H10T1/2 MSCs via activating the p38 mitogen-activated protein kinase (p38-MAPK)/Adenosine 5‘-monophosphate (AMP)-activated protein kinase α (AMPKα)/*drosophila* mothers against decapentaplegic protein (Smad1/5) pathway (Imran et al., 2017). Among these, the expressions of Uncoupling protein 1 (Ucp1), PR domain-containing 16 (Prdm16), and peroxlsome proliferator-activated receptor-γ coactlvator-1 (Pgc-1α) specific genes are dramatically elevated in brown adipocytes. Ucp1 is found only in thermogenic adipocytes (including brown and beige adipocytes) and serves as their morphological and functional signature (Pfeifer and Hoffmann, 2015). Additionally, CTS can boost the expression of certain genes, such as TNF Receptor Superfamily Member 9 (Cd137), Heat Shock Protein Family B (Small) Member 7 (Hspb7), cyclooxygenase-2 (Cox2), and Transmembrane Protein 26 (Tmem26) in beige adipocytes induced by the adipogenic hormone 0.5 mM IBMX, 1 μM Dexamethasone, and 10 μg/ml Insulin (MDI). Surprisingly, it can also drastically decrease the mRNA levels of two specific genes in white adipocytes, Phosphoserine Aminotransferase 1 (Psat1) and Resistin (Imran et al., 2017). Notably, activation of AMPK by CTS has been verified *in vitro* to differentiate C3H10T1/2 MSCs into brown adipocytes. However, *in vivo* research will need to be conducted in the future to further elucidate the regulatory mechanism through which CTS induces MSCs.

The identification of pathways and associated variables may result in developing new CTS-based obesity treatments in the future. CTS has demonstrated therapeutic potential in treating obesity, and additional research is necessary to fully understand CTS’s role in medicine.

## CTS and bone marrow mesenchymal stem cells

**FIGURE 2:**
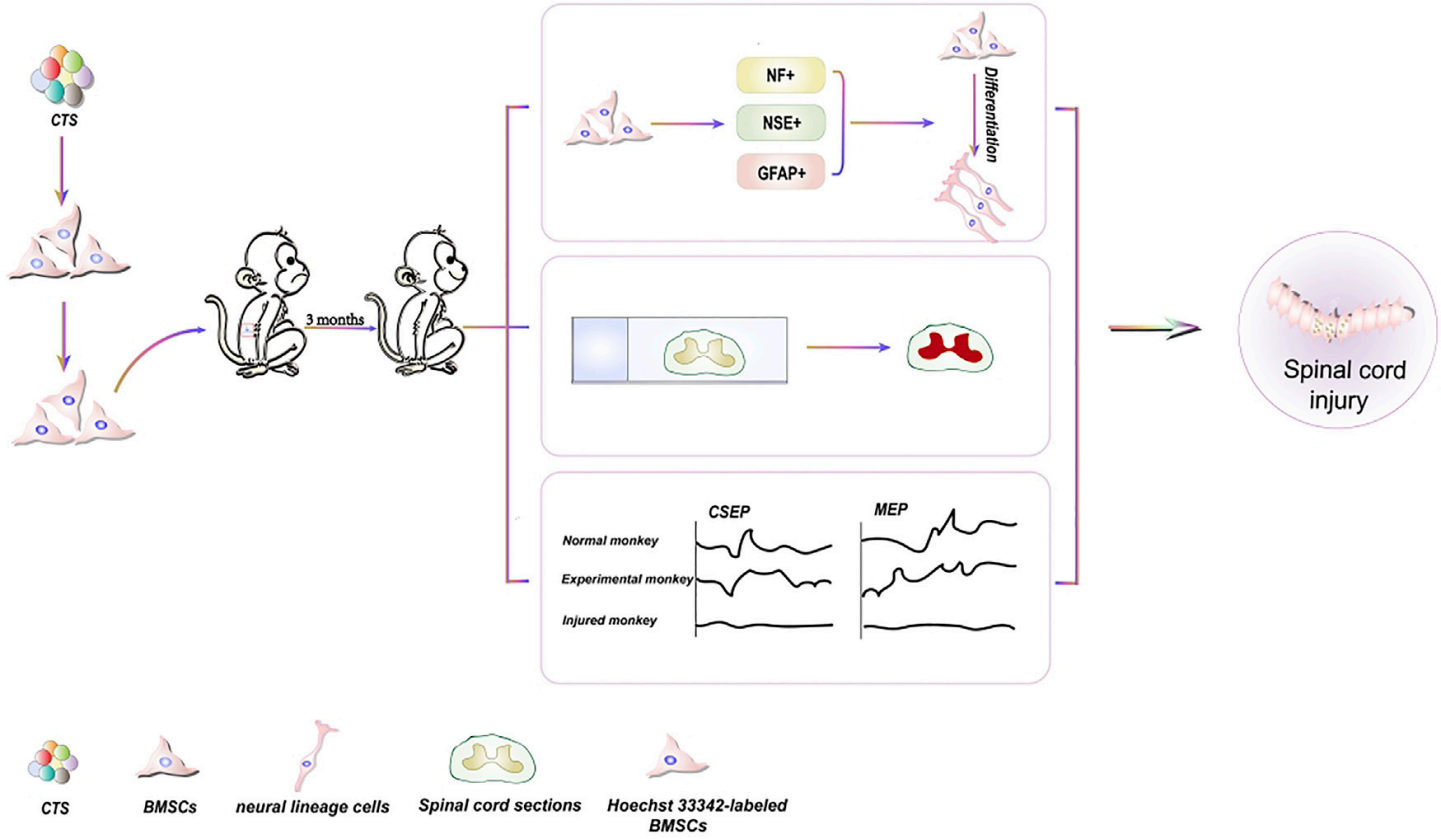
Schematic diagrams of the effects of CTS on differentiation of BMSCs for the treatment of spinal cord injury.

BMSCs are a type of pluripotent stem cell capable of self-replication and differentiation (Hao et al., 2019). BMSCs can be extracted from various tissues, including bone marrow, adipose tissue (Shanbhag et al., 2020), and umbilical cord blood (Contentin et al., 2020). BMSCs can develop into various cell types, including neurons (Hu et al., 2019), and offer distinct benefits in the treatment of spinal cord and nerve injuries. Spinal cord injury (SCI) is an incurable condition that results in the irreversible loss of motor, sensory, and sensory-motor capabilities below the level of the injury. Currently, there is no therapeutic intervention that guarantees complete recovery (Wilson et al., 2012; Chalfouh et al., 2020). CTS can induce the development of BMSCs into neural lineage cells (Figure 2), which makes them more suitable for spinal injury treatment (Deng et al., 2006). Additionally, CTS has been used to treat nervous system injuries (Hu et al., 2019; Li et al., 2019). It represents the therapeutic potential of CTS for SCI.

Neuron-specific enolase (NSE), glial fibrillary acidic protein (GFAP), and Neurofilament protein (NF) have been employed as markers for stem cell differentiation into neuronal lineage cells, and their expression ability can reflect stem cell development into neuronal cells (Singh et al., 2013). CTS-induced BMSCs are transplanted into spinal cord-injured monkeys. NF is discovered in 4% of Hoechst33342 stained cells. NSE and GFAP are detected in 5% of Hoechst33342 labeled cells. These findings reveal that CTS can cause BMSCs to differentiate into neuronal lineage cells. Simultaneously, the mRNA expression of GAD65 and GAD67 in the induced cells demonstrates that the differentiated cells can produce neurotransmitters. Furthermore, the monkey cortical somatosensory evoked potential (CSEP) and motor evoked potential (MEP) with SCI reverted to normal, and spinal cord tissue samples are effectively stained with hematoxylin and eosin, demonstrating spinal cord healing and regeneration (Deng et al., 2006).

Unfortunately, the specific mechanisms underlying the differentiation of BMSCs into neural lineage cells induced by CTS are still not fully illustrated yet. As a phosphoinositide 3-kinase (PI3K) inhibitor, LY294002 can stimulate the differentiation of BMSCs into neural cells (Wang et al., 2012). This study shows that inhibiting PI3K/v-akt murine thymoma viral oncogene homolog (AKT) could be a useful mechanism for stem cell research in treating neurological illnesses. A useful tool for tiny compounds that block routes (Wang et al., 2012). CTS plays a vital role by suppressing PI3K/AKT, such as reducing nerve pain after surgery and having anti-stroke properties (Zhu et al., 2017; Zhang et al., 2019). However, it is unclear whether CTS can stimulate BMSC proliferation and differentiation via modulating PI3K/AKT pathway expression.

## CTS and neural stem/progenitor cells

NSCs are mostly observed in the adult central nervous system as a continuous supply of nerve cells (Draijer et al., 2019). NSCs can self-renewal and are pluripotent. As a result, NSCs can generate all neuroectodermal lineages in a manner appropriate for locations and developmental stages (Kahroba et al., 2021). Furthermore, NSCs can spontaneously develop into neurons, astrocytes, or oligodendrocytes (Wang et al., 2015b; Draijer et al., 2019). NSCs can be stimulated to proliferate, migrate, and differentiate in response to the central nervous system and pathological injury (Kernie and Parent, 2010; Sun, 2014). This, in turn, stimulates neurogenesis (Laterza et al., 2017; Wu et al., 2017). Unfortunately, due to its low cell count, endogenous neurogenesis does not fully promote repairing and regenerating nerve-damaged neurons, particularly in severe nerve injury such as stroke (Kernie and Parent, 2010). As a result, NSC transplantation for the treatment of severe neurodegenerative diseases may be a viable alternative to compensating for abnormalities in the endogenous neural repair mechanism (Boese et al., 2018).

### The regulatory role of CTS in NSC proliferation and differentiation

**FIGURE 3:**
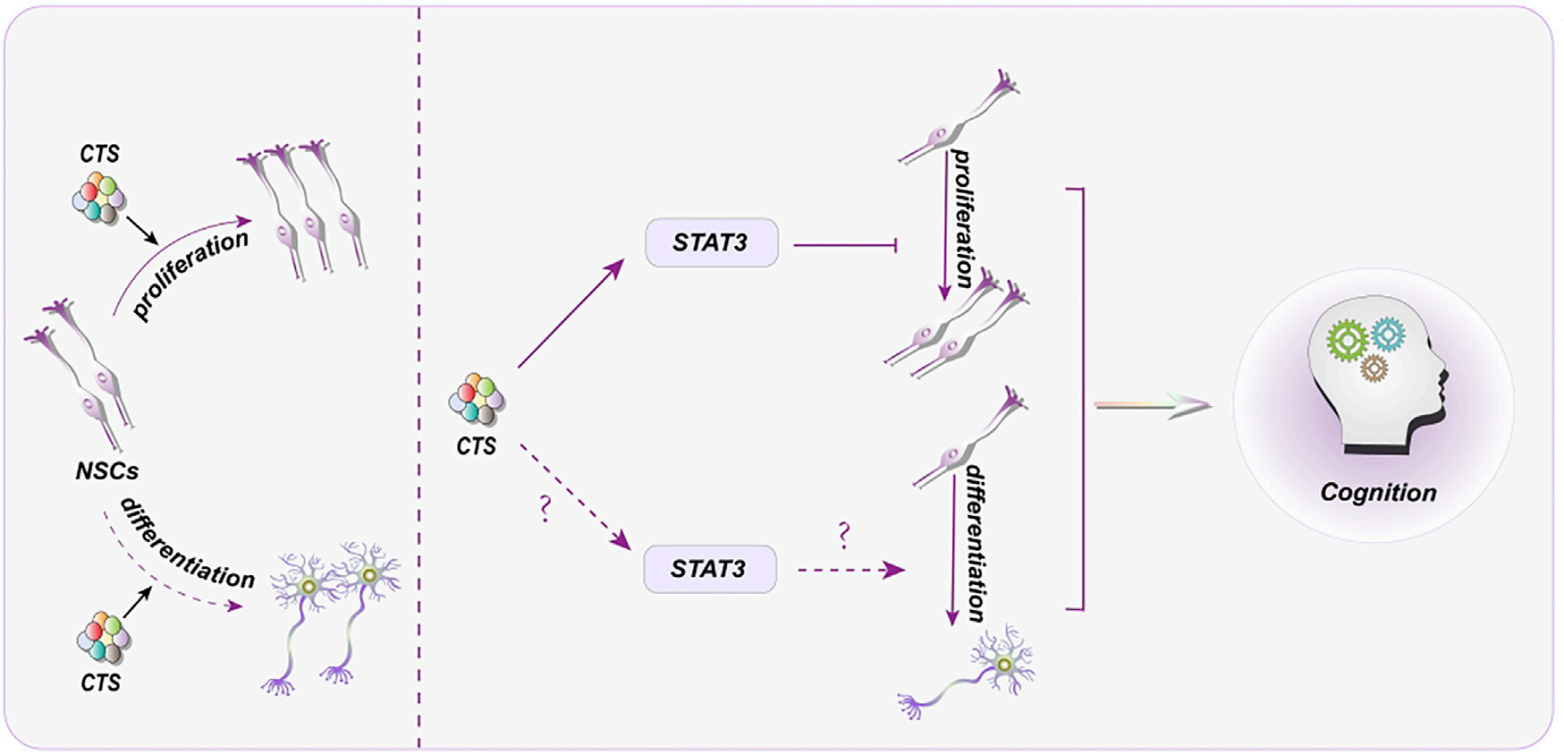
Representative scheme illustrating the potential mechanisms underlying the CTS regulation in proliferation and differentiation of NSCs for the treatment of cognitive disorder.

By inhibiting Signal transducer and activator of transcription 3 (STAT3), CTS can prevent the proliferation of human embryonic stem cells-derived NSCs (Zhang et al., 2016a) (Figure 3). As a STAT3 inhibitor, CTS can reduce CSC proliferation as well (Xiong et al., 2018). It will aid future research into the interaction between CTS and stem cells and provide insight into whether the same target may be employed in diverse disorders.

Despite the intense interest in the impact of CTS on NSC proliferation and differentiation, the precise mechanisms behind this regulatory role remain unclear. Thus, how to control NSC and its potential mechanisms induced by CTS may provide a permissive milieu for adult neurogenesis in neurological illnesses. By inhibiting STAT3, CTS can prevent the proliferation of human NSCs. Intriguingly, blocking STAT3 may also boost NSC proliferation (Ma et al., 2018). It is worth investigating whether STAT3 inhibition has distinct effects on NSCs derived from different sources or whether varying concentrations of CST affect NSCs. Similarly, STAT3 plays a critical function in NSC differentiation (Figure 3). STAT3 inhibition can minimize the severity of spinal cord injury (Cui et al., 2017), suppress astrocyte production, and increase NSC neurogenesis (Cao et al., 2010). These findings strongly imply that CTS, via decreasing STAT3, may play a role in neurogenesis.

Despite an accumulating body of research indicates the impact of Chinese herbal monomers on NSCs (Wang et al., 2021d; Wang et al., 2021b), the precise mechanisms are still not fully understood yet. There is no doubt that STAT3 has been a hot topic in the proliferation and differentiation of NSCs (Zyuz’kov et al., 2020; Li et al., 2021). CTS can bind to the SH2 domain of STAT3 and inhibit the phosphorylation of STAT3, preventing STAT3 from forming dimer and make STAT3 unable to serve a function (Shin et al., 2009). In brief, STAT3 is a bridge between CTS and NSCs, which is conducive to the further study of NSCs by CTS.

### The regulatory role of CTS in NPC apoptosis

**FIGURE 4:**
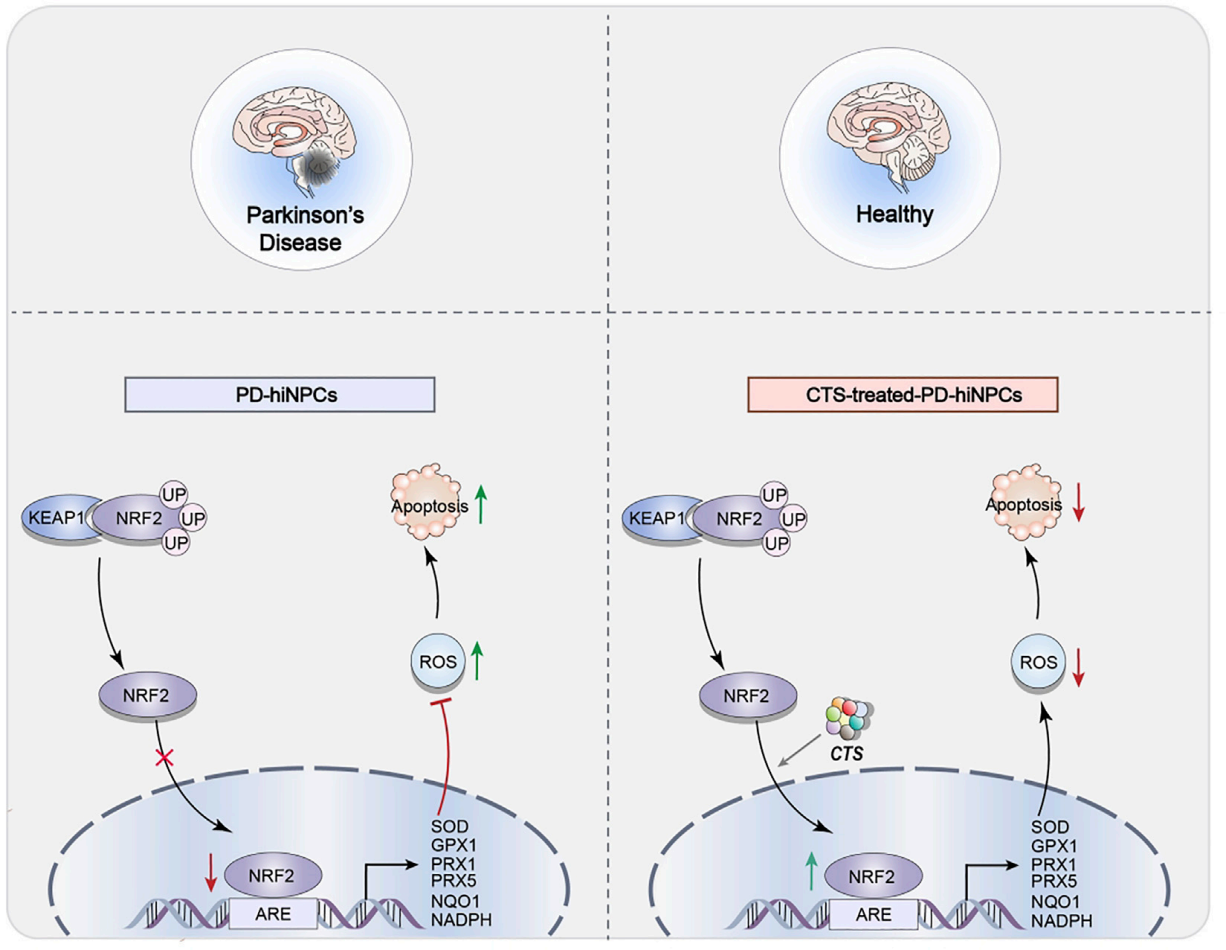
Representative scheme illustrating CTS’s inhibitory role in the apoptosis of NPCs by regulating the NRF2 signaling pathway for the treatment of parkinson’s disease.

CTS can inhibit the apoptosis of Parkinson’s disease patient-derived human-induced neuronal progenitor cells (PD-hiNPCs) by restoring the membrane potential availability in PD-hiNPCs, reducing the levels of total ROS and mitochondrial ROS, and down-regulating the expression of apoptotic protein caspase-3 (Lee et al., 2020). ROS have been recognized as an important factor in Parkinson’s disease (Hemmati-Dinarvand et al., 2019). CTS can exert antioxidant effects by activating the transcription of nuclear factor erythroid 2-related factor 2 (NRF2) signaling pathway (Figure 4). Among them, CTS can boost the expression of certain genes, such as superoxide dismutase 1 (SOD1), peroxiredoxin 1 (PRX1), peroxiredoxin 5 (PRX5), glutathione peroxidase (GPX1) and NAD(P)H quinone dehydrogenase 1 (NQO1) in PD-hiNPCs (Lee et al., 2020). These results provide evidence for CTS as a treatment for PD.

Stem cell therapy has shown significant promise in treating neurodegenerative disorders such as Alzheimer’s and Huntington’s disease (Duncan and Valenzuela, 2017). Simultaneously, CTS has made strides in Alzheimer’s disease research (Mei et al., 2009; Maione et al., 2018). However, the therapeutic effect of transplantation of NSCs pretreated with CTS *in vitro* is unclear. Therefore, further studies are needed to clarify how CTS mediates the proliferation and differentiation of NSCs. These findings will provide the basis for new therapeutic strategies for the treatment of neurodegenerative diseases.

## CTS and cancer stem cells

**FIGURE 5:**
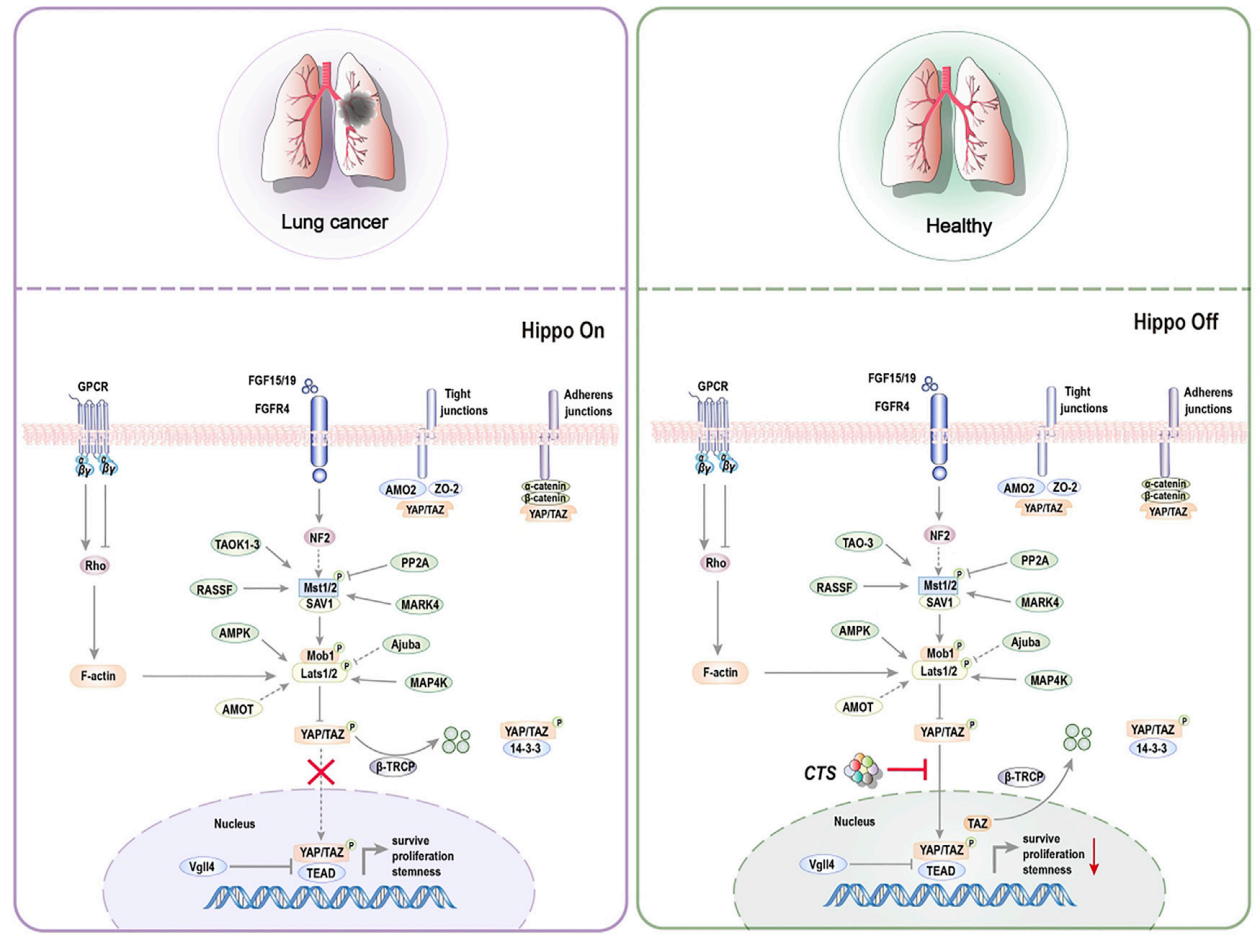
Representative scheme illustrating CTS’s inhibitory role in the proliferation of NSCL CSCs by regulating the Hippo signaling pathway for the treatment of non-small cell lung cancer.

**FIGURE 6:**
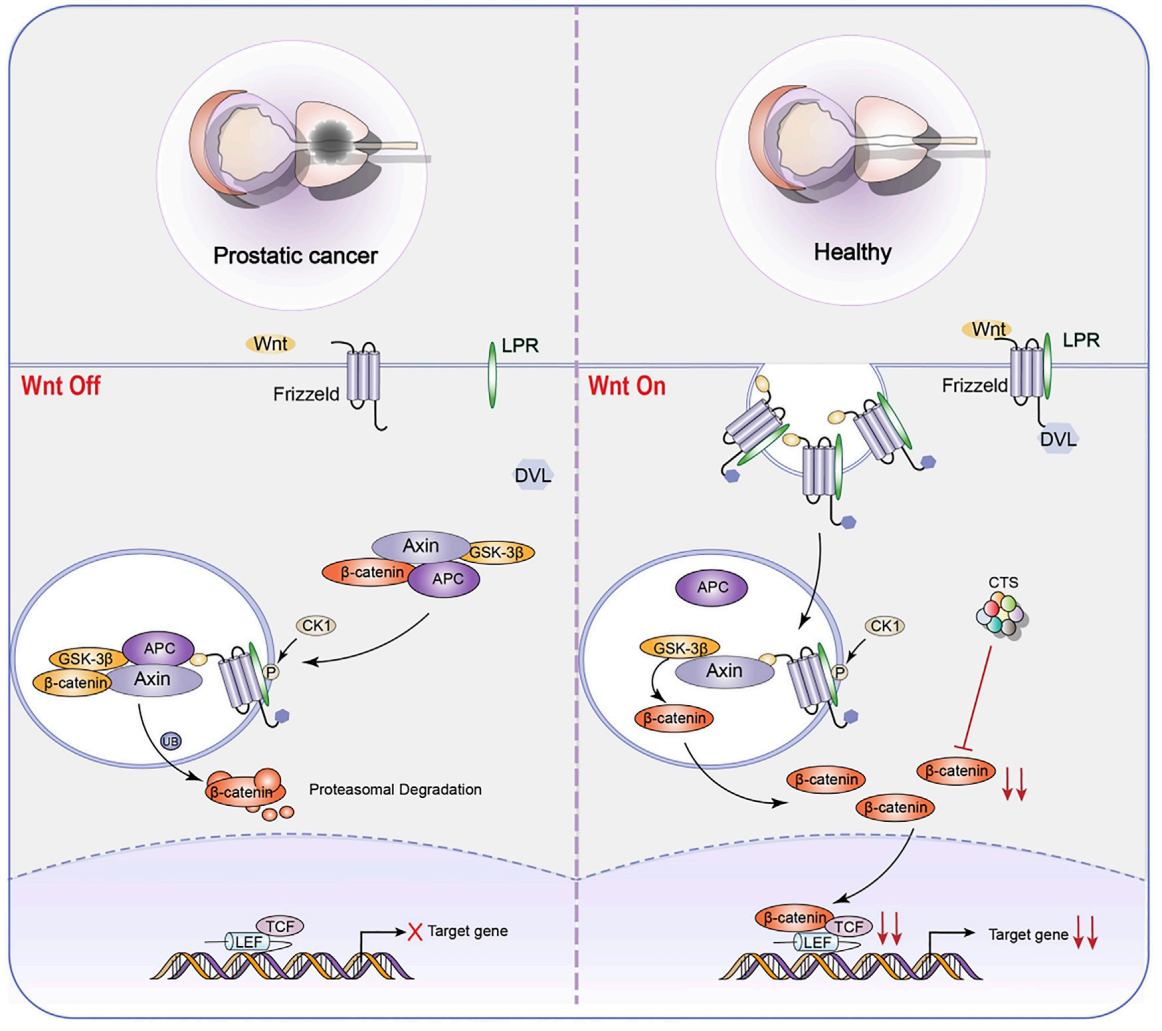
Representative scheme illustrating CTS’s inhibitory role in the proliferation of LNCaP TICs by regulating the Wnt signaling pathway for the treatment of prostatic cancer.

Tumor-initiating cells (TICs), also known as CSCs, are the primary causes of recurrence, metastasis, and poor prognosis in many types of cancer. As a result, it is thought to be the source of tumorigenesis (Li et al., 2018b; Atashzar et al., 2020). CSCs are more resistant to chemotherapy and radiation treatments, posing a catastrophic dilemma in cancer treatment (Hoey et al., 2009; Vermeulen et al., 2010; Takebe et al., 2015). Stem cells may be the source of all tumor cells in malignant tumors and the primary cause of drug resistance, tumor recurrence, metastasis, and poor prognosis (Maccalli et al., 2018). According to research, targeting or relieving CSCs can restrict tumor formation and progression and minimize treatment resistance, hence preventing tumor progression (Mai et al., 2017; Sun et al., 2017). At the moment, CTS is vital in limiting the proliferation of tumor stem cells (Ding et al., 2016). It has shown promise in inhibiting non-small cell lung cancer stem cells (NSCL CSCs) (Figure 5) and prostate initiating cells (Figure 6).

The aberrant activation of the Hippo signaling pathway and Yes-associated protein (YAP)/transcriptional co-activator with PDZ-binding motif (TAZ)-transcriptional enhancer associate domain (TEAD) has been linked to cancer, making this pathway an appealing target for therapeutic intervention (Arthur et al., 2009). CTS controls the ectopic movement of TAZ from the nucleus to the cytoplasm, lowering the stem cell characteristics of NSCL CSCs (Jin et al., 2020). The Hippo pathway is divided into two sections. Macrophage Stimulating one and Macrophage Stimulating 2 (MST1/2), Mitogen-Activated Protein four Kinases (MAP4K), and Large Tumor Suppressor Kinase one and Large Tumor Suppressor Kinase 2 (LATS1/2) are the key inhibitory kinase modules, and YAP/TAZ and TEAD are transcription modules. When Hippo pathway is activated, LATS1/2 phosphorylates YAP/TAZ directly and inhibits nuclear YAP/TAZ through 14-three to three mediated cytoplasmic retention and ubiquitination-mediated proteasome and autolysosome degradation. Vestigial Like Family Member 4 (VGLL4) inhibits TEAD transcription activity. When Hippo pathway is closed, YAP/TAZ is dephosphorylated and delivered to the nucleus, where it combines with the transcription factor TEAD, allowing target gene transcription to contribute to cell proliferation (Park et al., 2018). CTS inhibits the expression of TAZ target genes Connective tissue growth factor (CTGF), Transcriptional Intermediary Factor 1 (TIF-1), and Mothers Against Decapentaplegic Homolog 2 (Smad2) but does not affect YAP. CTS, however, does not impact the phosphorylation of TAZ’s upstream gene LATS1/2 but instead work directly on TAZ, regulating TAZ translocation from the nucleus to the cytoplasm. Furthermore, CTS decreases the expression of NSCL CSCs specific markers octamer-binding transcription factor 4 (Oct4), Nanog, and Aldehyde Dehydrogenase one Family (ALDH1) mRNA levels while increasing the expression of Cyclin Dependent Kinase 3 (CDK3), Integrin Subunit Alpha X (CD11c), and High affinity immunoglobulin gamma Fc receptor I (CD64) mRNA levels (Jin et al., 2020).

CTS can reduce the proliferation of NSCL CSCs by reducing Hippo signaling pathway and the proliferation of LNCaP TICs by inhibiting Wnt signaling pathway (Zhang et al., 2016b). The Wingless-Int1 (Wnt)/β-catenin signaling pathway has been demonstrated to be intricately related to prostatic CSCs (Schneider and Logan, 2018). Cell fate determination has been linked to abnormalities in the regulation of Wnt/β-catenin pathway. The Wnt/β-catenin pathway could be activated in several ways, including the binding of Wnt protein to members of Frizzled family of receptors. When Wnt pathway is engaged, GSK3β may phosphorylate freshly synthesized β-catenin protein, preventing them from entering the nucleus and playing a role. When GSK3β is inactive, β-catenin cyclic protein accumulates and is transported to the nucleus, activating the downstream target genes, influencing cell self-renewal and proliferation (Valkenburg et al., 2011). The most notable property is CTS’s ability to suppress β-catenin protein expression. As a result, stem cell growth and stemness are inhibited. Additionally, CTS dose-dependently decreased the protein expression of the stem cell genes Nanog, SRY-Box Transcription Factor 2 (Sox2), and Oct4.

Intriguingly, CTS decreases C-X-C Motif Chemokine Receptor 4 (CXCR4) mRNA and protein levels. Furthermore, CTS can regulate biological activities associated with the function of stromal cell derived factor 1 (SDF1)/CXCR4 axis, such as migration and metastasis (Zhang et al., 2016b). This demonstrates that CTS may be a promising small molecule therapeutic candidate for suppressing LNCaP TIC growth.

Finally, CTS has the potential to be a good anticancer drug since it can suppress the proliferation of diverse CSCs. It is hoped that future research will delve deeper into the past association between CTS and CSCs.

## Conclusion and future perspectives

This review summarizes current evidences on the role and impact of CTS in various stem cells and provides a new perspective on the prevention and treatment of high-incidence diseases. More importantly, CTS exhibits great potential in various high-incidence diseases and influences the cell proliferation/self-renewal and differentiation of stem cells. It is critical to investigate the potential mechanism of CTS in the proliferation and differentiation of enormous stem cells, as this may provide further opportunities and references for future CTS research. So far, research on the impact of CTS on stem cells remains in its early phases, and it is unclear how CTS affects stem cell destiny regulation and integration. The effect of CTS on stem cells progresses to *in vivo* research, which will eventually alter disease treatment. Although significant progress has been made in understanding the role of CTS in stem cell proliferation and destiny regulation, we are only now beginning to understand its significance in stem cell development. Stem cells provide a wonderful platform for understanding CTS function and a unique possibility for developing new therapeutics and treatments for high-incidence diseases. It is critical to focus on stem cell biology and molecular mechanisms in future studies. As a result, additional research into the effects of CTS on stem cells can assist the medical community in realizing its therapeutic potential through stem cell treatment.

The introduction of several compound drugs, including CTS, gives us complete confidence in the clinical prospects of CTS. The ability of CTS to cross the blood-brain barrier fulfills the FDA’s quantitative drug analysis standard (Wang et al., 2022a). However, it should be recognized that CTS carries potential hazards. CTS has been demonstrated to have pharmacological toxicity in both *in vivo* zebrafish research (Wang et al., 2021a) and *in vitro* cell experiments (Wu et al., 2016; Kim et al., 2017), which is the most important problem for CTS to be solved. Furthermore, CTS has poor water solubility, poor oral absorption, low bioavailability, and high photosensitivity, which are some of the factors limiting its development (Wang et al., 2022c; Zhang et al., 2022), but there have been many studies to improve CTS bioavailability through spray formulations (Wang et al., 2022c), nanoparticles (Zhang et al., 2022), and nano-emulsions (Chengxi et al., 2019). CTS requires large-scale, randomized, double-blind, and controlled clinical trials to validate the safety and increase clinical efficacy. Although progress has been made in understanding the role of CTS in high-incidence diseases, what is urgently needed is to explore its impact on different stem cells and future therapeutic potential for various diseases.

## Glossary

CTS: Cryptotanshinone
S. miltiorrhiza: Salvia miltiorrhiza Bunge
C3H10T1/2 MSCs: C3H10T1/2 mesenchymal stem cells
BMSCs: bone marrow mesenchymal stem cells
NSCs/NPCs: neural stem/progenitor cells
CSCs: cancer stem cells
AMPK: amp-activated protein kinase
p38-AMPK: p38 mitogen-activated protein kinase
AMPKα: Adenosine 5‘-monophosphate (AMP)-activated protein kinase α
Smad1/5: drosophila mothers against decapentaplegic protein
BAT: brown adipose tissue
Ucp1: Uncoupling protein 1
Prdm16: PR domain-containing 16
Pgc-1α: peroxlsome proliferator-activated receptor-γ coactlvator-1
Cd137: TNF Receptor Superfamily Member 9
Hspb7: Heat Shock Protein Family B (Small) Member 7
Cox2: cyclooxygenase-2
Tmem26: Transmembrane Protein 26
MDI: 0.5 mM IBMX, 1 μM Dexamethasone and 10 μg/ml Insulin
Psat1: Phosphoserine Aminotransferase 1
SCI: Spinal cord injury
NSE: Neuron specific enolase
GFAP: glial fibrillary acidic protein
NF: Neurofilament protein
BMSCs: Bone marrow mesenchymal stem cells
CSEP: cortex somatosensory evoked potential
MEP: motor evoked potential
PI3K/AKT: phosphoinositide 3-kinase/v-akt murine thymoma viral oncogene homolog
STAT3: Signal transducer and activator of transcription 3
PD-hiNPCs: Parkinson’s disease patient-derived human-induced neuronal progenitor cells
NRF2: nuclear factor erythroid 2-related factor 2
SOD1: superoxide dismutase 1
PRX1: peroxiredoxin 1
PRX5: peroxiredoxin 5
GPX1: glutathione peroxidase
NQO1: NAD(P)H quinone dehydrogenase 1
TICs: tumor-initiating cells
NSCL: non-small cell lung
CSCs: cancer stem cells
YAP: Yes-associated protein
TAZ: transcriptional co-activator with PDZ-binding motif
TEAD: transcriptional enhancer associate domain
MST1/2: Macrophage Stimulating 1 and Macrophage Stimulating 2
MAP4K: Mitogen-Activated Protein Kinase Kinase Kinase Kinase
LATS1/2: Large Tumor Suppressor Kinase 1 and Large Tumor Suppressor Kinase 2
VGLL4: Vestigial Like Family Member 4
CTGF: Connective tissue growth factor
TIF-1: Transcriptional Intermediary Factor 1
Smad2: Mothers Against Decapentaplegic Homolog 2
Oct4: octamer-binding transcription factor 4
ALDH1: Aldehyde Dehydrogenase 1 Family
CDK3: Cyclin Dependent Kinase 3
CD11c: Integrin Subunit Alpha X
CD64: High affinity immunoglobulin gamma Fc receptor I
Wnt/β-Catenin: Wingless-Int1/β-Catenin
Sox2: SRY-Box Transcription Factor 2
CXCR4: C-X-C Motif Chemokine Receptor 4
SDF1: stromal cell derived factor 1
