# Supplementary figures and images for: Novel perspectives on the therapeutic role of cryptotanshinone in the management of stem cell behaviors for high-incidence diseases
Source: Front Pharmacol. 2022 Aug 15;13:971444. doi: 10.3389/fphar.2022.971444 (PMC9420941; doi:10.3389/fphar.2022.971444)

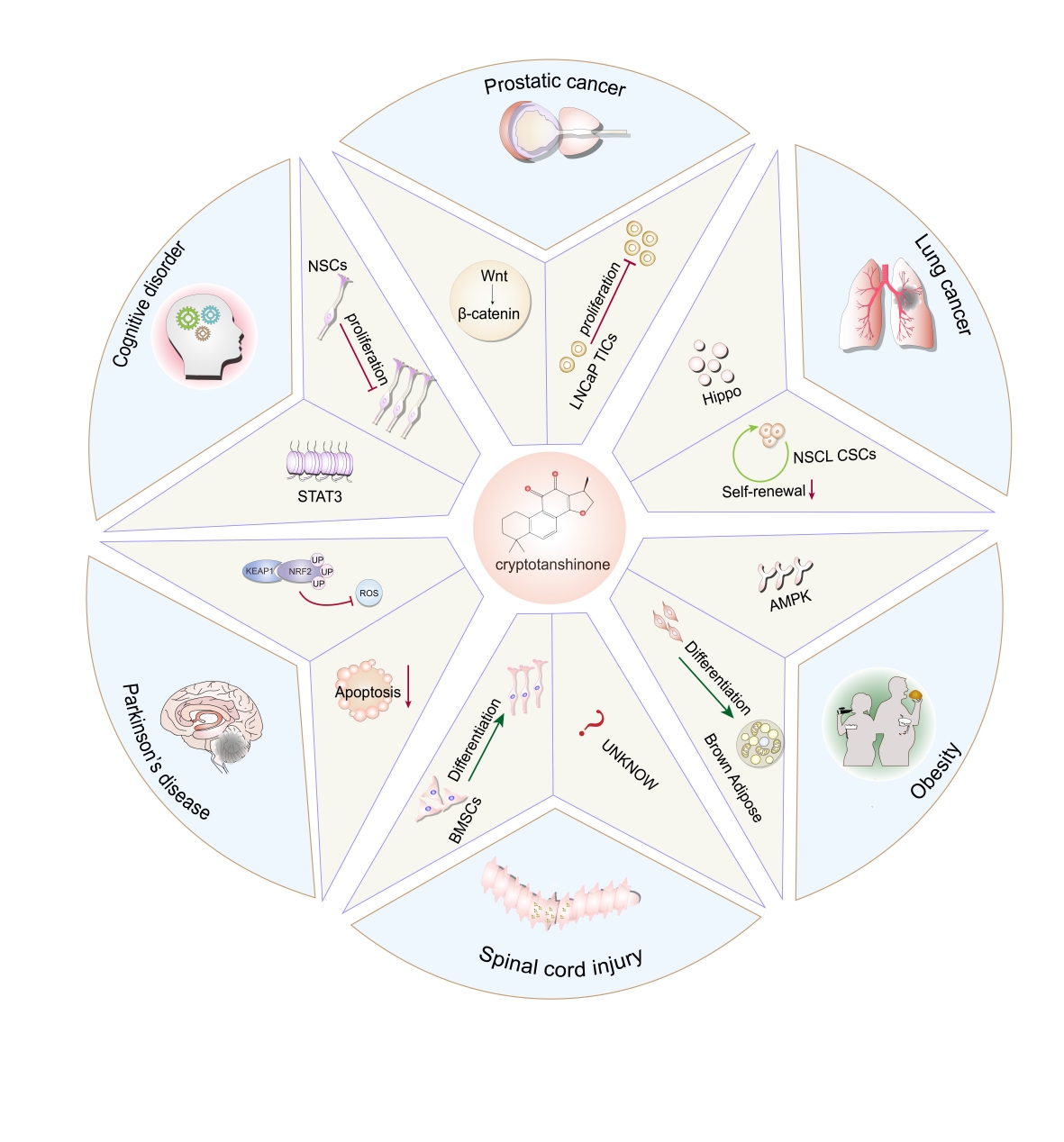

Supplement: Supplementary file 1 [file Image1.JPEG]
